# Five BSC members selected in new cohort of New Cornerstone Investigators

**DOI:** 10.52601/bpr.2025.250902

**Published:** 2025-12-31

Five distinguished members of the Biophysical Society of China (BSC) have been named to the third cohort of the New Cornerstone Investigator Program, according to the list unveiled by the New Cornerstone Science Foundation on Nov 24, 2025.

Among the 35 scientists recognized this year, the BSC selectees include Yanyi Huang, vice-president of the Single-cell Multiomics Academic Subgroup and vice-president of the Microfluidic System Academic Subgroup, whose research focuses on single-cell multiomics, microfluidic technology, and high-throughput genomic sequencing; Peilong Lu, a committee member of the Molecular Biophysics Academic Subgroup, specializing in protein design, particularly the *de novo* design of membrane protein antagonists and functional membrane proteins; Xiangxi Wang, secretary-general of the BSC, working in virology and structural biology, with a focus on the precise observation and design of biomacromolecules; Yanli Wang, a member of the BSC, focusing on the structure and function of RNA interference-related proteins and the mechanisms of CRISPR/Cas systems; and Li Yu, a committee member of the Membrane Biophysics Academic Subgroup, who discovered the biological phenomenon of migrasomes and is developing a migrasome-based drug delivery platform.

Their selection demonstrates the strong scientific leadership and innovative capacity of the biophysics community in China and highlights the BSC’s ongoing commitment to fostering excellence in frontier research.

The New Cornerstone Investigator Program is an innovative philanthropic funding initiative designed to support fundamental research with a focus on originality and free exploration. In 2022, Tencent committed 10 billion yuan ($1.4 billion) over 10 years to provide long-term and stable support for outstanding scientists working toward groundbreaking innovations.

The program supports creative scientists engaged in high-risk, exploratory basic research projects, aiming to empower them to raise major scientific questions, push disciplinary boundaries and drive original breakthroughs.

It encompasses two broad fields: Mathematics & Physical Sciences and Biological & Biomedical Sciences, and strongly encourages interdisciplinary research. The Mathematics and Physical Sciences category covers areas of mathematics, physics, chemistry and theoretical computer science that do not have immediate industrial application possibilities.

Two funding tracks are offered: experimental and theoretical. Each funding cycle lasts five years, providing 25 million yuan per investigator for experimental research and 15 million yuan for theoretical research. Recipients may apply for renewal upon completion of their terms.

To date, three cohorts have been selected, with a total of 139 exceptional scientists recognized as New Cornerstone Investigators.

**Table:** 

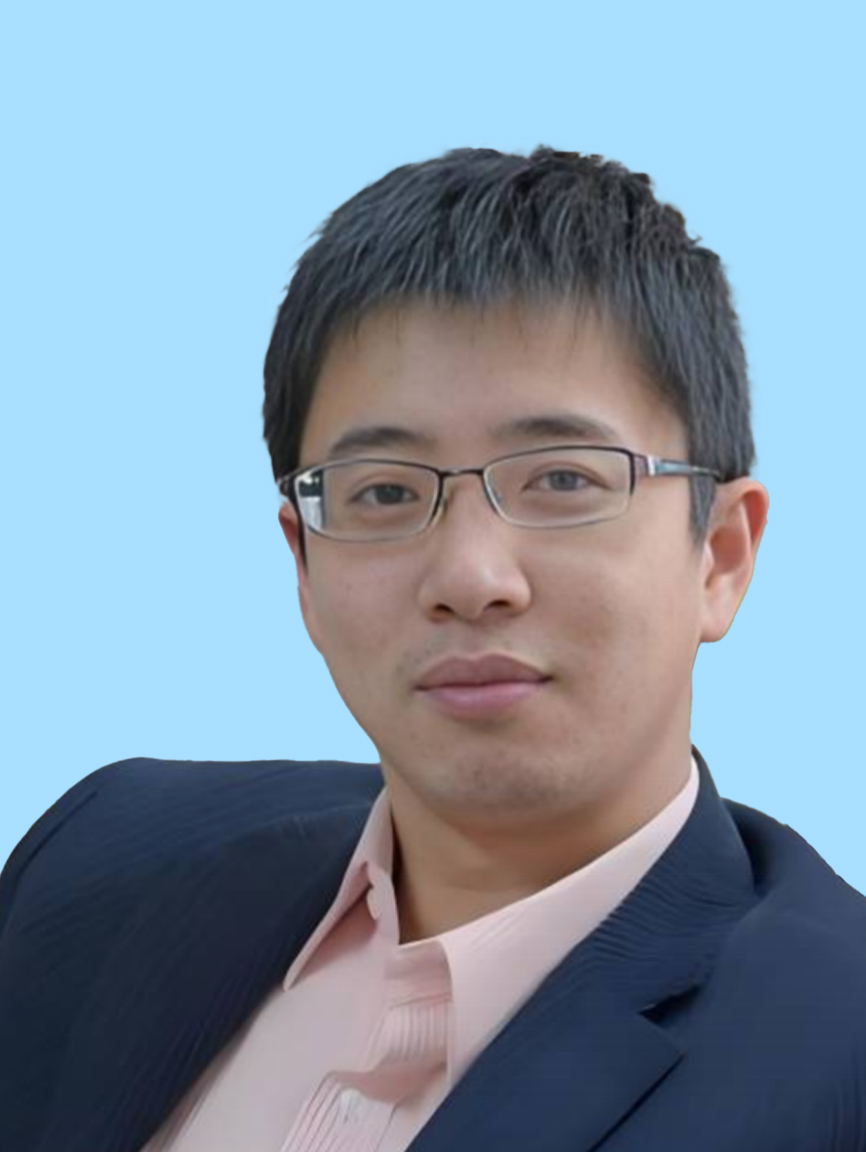	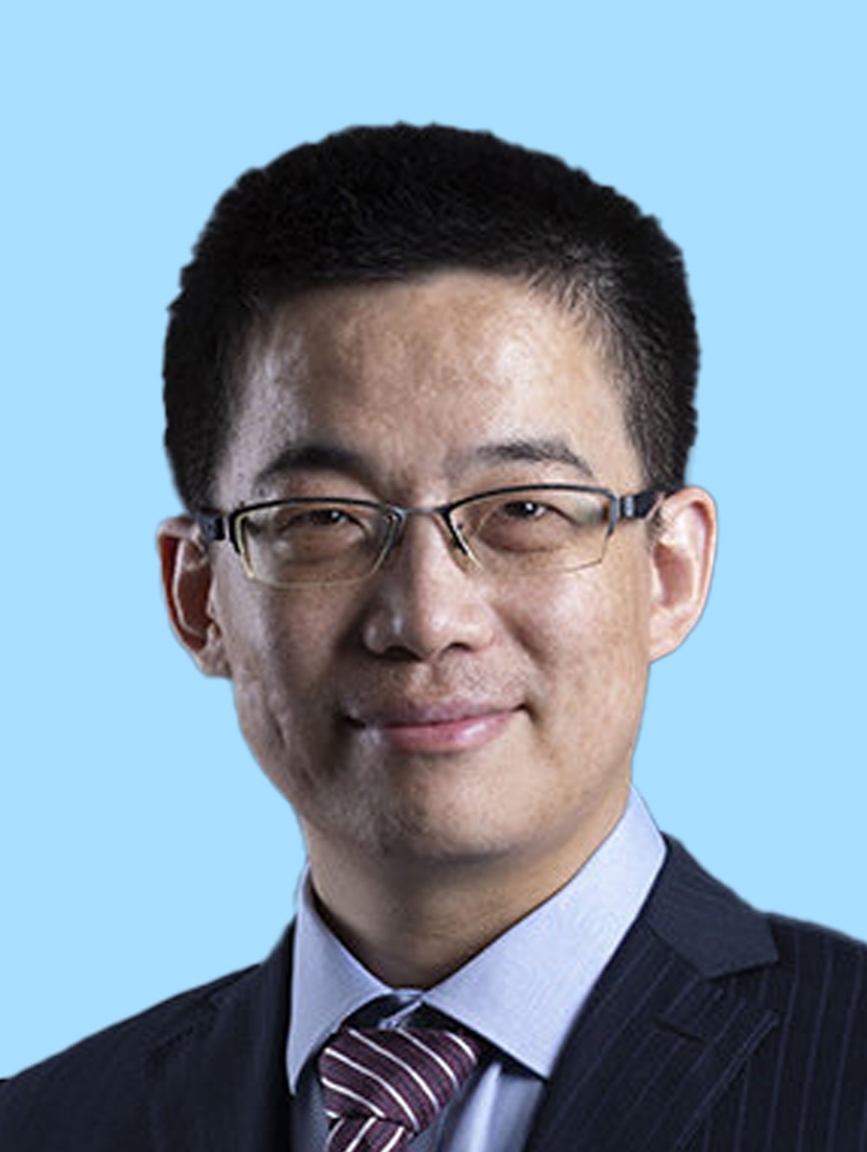	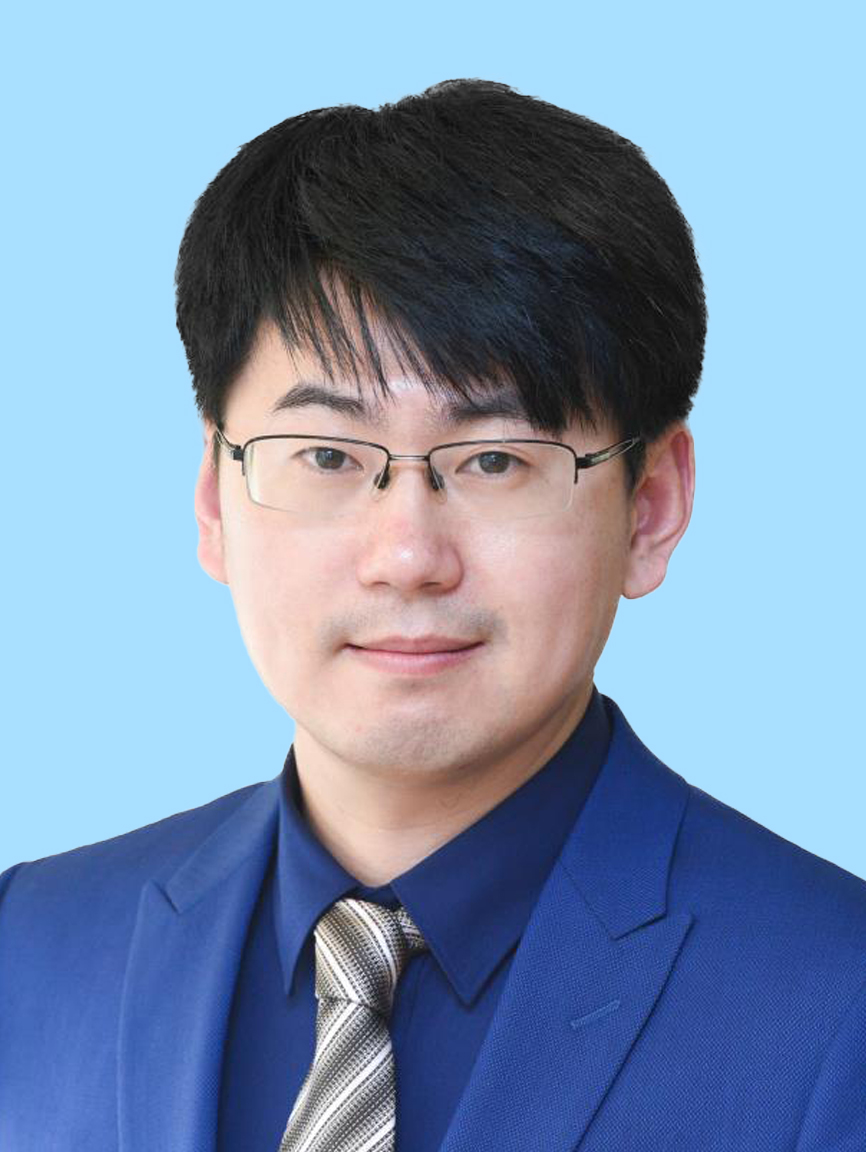	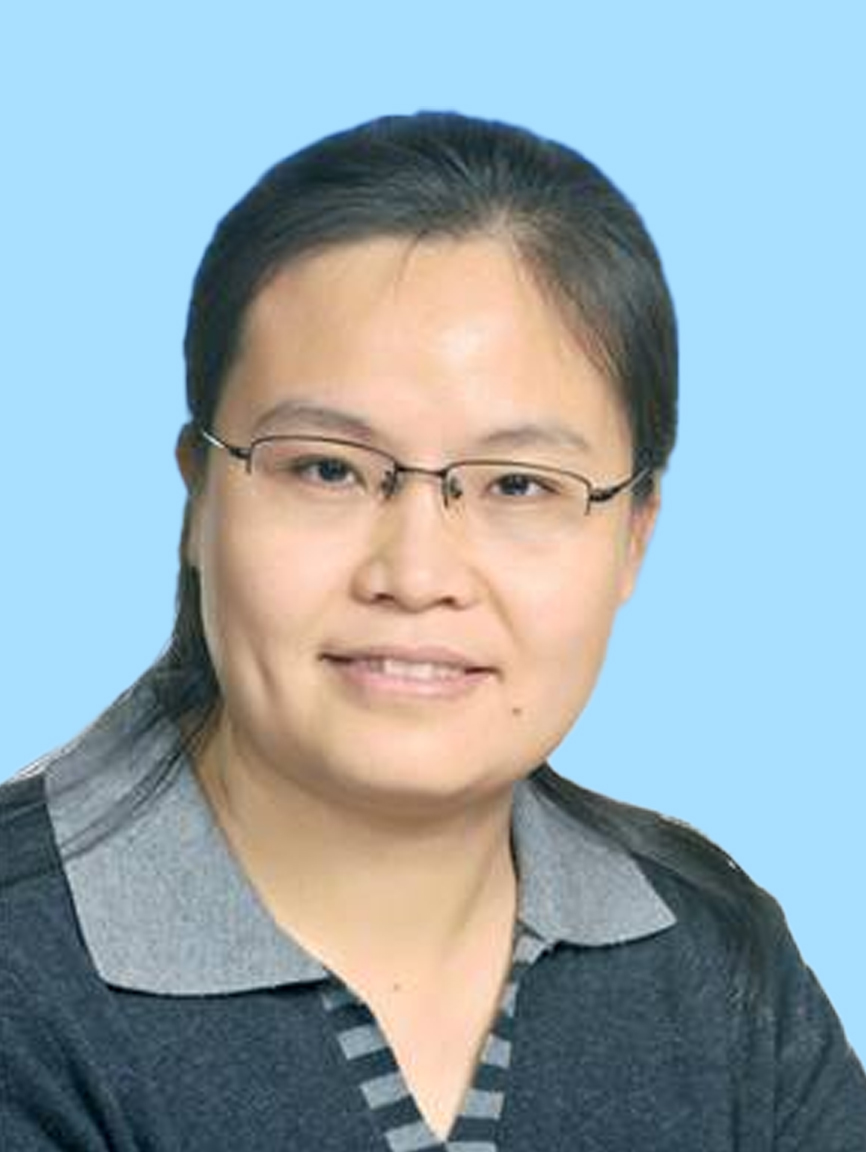	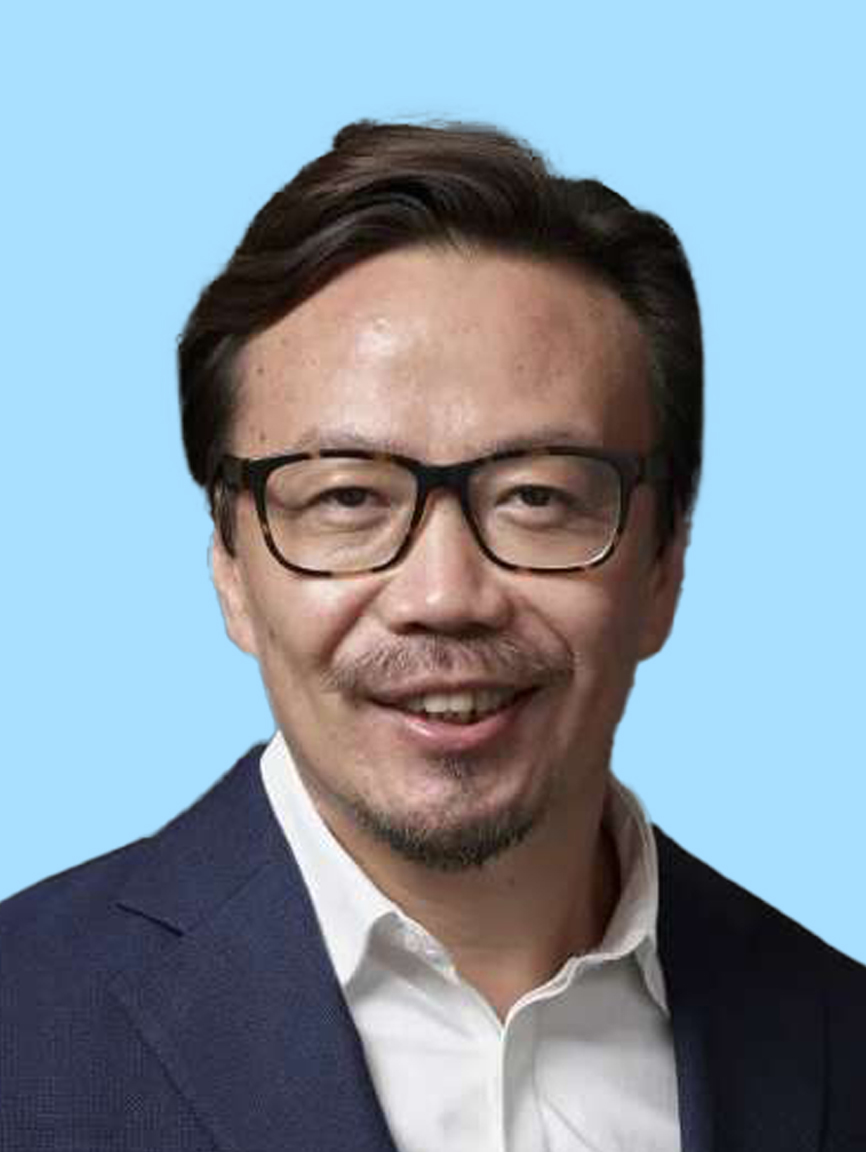
Yanyi Huang	Peilong Lu	Xiangxi Wang	Yanli Wang	Li Yu

## Conflict of interest

 declare that they have no conflict of interest.

